# Thyroid hormone reduces PCSK9 and stimulates bile acid synthesis in humans[S]

**DOI:** 10.1194/jlr.M051664

**Published:** 2014-11

**Authors:** Ylva Bonde, Olof Breuer, Dieter Lütjohann, Stefan Sjöberg, Bo Angelin, Mats Rudling

**Affiliations:** * Metabolism Unit, Department of Endocrinology, Metabolism, and Diabetes, and KI/AZ Integrated CardioMetabolic Center, Department of Medicine; † Molecular Nutrition Unit, Center for Innovative Medicine, Department of Biosciences and Nutrition; § Karolinska Institute at Karolinska University Hospital Huddinge, S-14186 Stockholm, Sweden; Karo Bio AB, Novum, S-14186 Stockholm, Sweden; ** Institute of Clinical Chemistry and Clinical Pharmacology, University Clinics Bonn, D-53105 Bonn, Germany

**Keywords:** lipoproteins/metabolism, cholesterol 7alpha-hydroxylase, cholesterol/absorption, bile acids and salts/biosynthesis, fibroblast growth factor, fibroblast growth factor 19, fibroblast growth factor 21, proprotein convertase subtilisin/kexin type 9, eprotirome, drug therapy/hypolipidemic drugs

## Abstract

Reduced plasma LDL-cholesterol is a hallmark of hyperthyroidism and is caused by transcriptional stimulation of LDL receptors in the liver. Here, we investigated whether thyroid hormone (TH) actions involve other mechanisms that may also account for the reduction in LDL-cholesterol, including effects on proprotein convertase subtilisin/kexin type 9 (PCSK9) and bile acid synthesis. Twenty hyperthyroid patients were studied before and after clinical normalization, and the responses to hyperthyroidism were compared with those in 14 healthy individuals after 14 days of treatment with the liver-selective TH analog eprotirome. Both hyperthyroidism and eprotirome treatment reduced circulating PCSK9, lipoprotein cholesterol, apoB and AI, and lipoprotein(a), while cholesterol synthesis was stable. Hyperthyroidism, but not eprotirome treatment, markedly increased bile acid synthesis and reduced fibroblast growth factor (FGF) 19 and dietary cholesterol absorption. Eprotirome treatment, but not hyperthyroidism, reduced plasma triglycerides. Neither hyperthyroidism nor eprotirome treatment altered insulin, glucose, or FGF21 levels. TH reduces circulating PSCK9, thereby likely contributing to lower plasma LDL-cholesterol in hyperthyroidism. TH also stimulates bile acid synthesis, although this response is not critical for its LDL-lowering effect.

Thyroid hormone (TH) is a potent regulator of multiple metabolic pathways by interaction with TH nuclear receptors in various tissues (1–3). Lipoprotein metabolism is strongly influenced by TH, and dyslipidemia is common in thyroid disorders (4). Reduced plasma LDL-cholesterol is a hallmark of hyperthyroidism and is caused by increased transcription of LDL receptors (LDLRs) in the liver. In rodents, TH stimulates processes that contribute to elimination of cholesterol from the body, including the conversion of cholesterol into bile acids (5) and biliary secretion of bile acids and cholesterol (6). TH also diminishes intestinal absorption of dietary cholesterol (7) and stimulates cholesterol synthesis (5). The importance of these mechanisms for lowering LDL-cholesterol in humans is somewhat unclear, as is the possible involvement of novel regulators of lipid metabolism such as proprotein convertase subtilisin/kexin type 9 (PCSK9) (8) and fibroblast growth factor (FGF) 19 and 21 (9). The aim of this study was therefore to further characterize the effects of TH on cholesterol and lipoprotein metabolism in humans. For this purpose, two models of exposure to TH were used: *a*) patients with hyperthyroidism before and after clinical normalization, and *b*) healthy volunteers treated for 14 days with a liver-selective TH analog, eprotirome (10, 11).

## MATERIALS AND METHODS

### Subjects and study design

The first study (a) included 16 women and 4 men who had been referred to our outpatient unit due to hyperthyroidism. They were between 18 and 73 years old (mean ± SD, 46 ± 14 years) with serum levels of thyroid stimulating hormone (TSH) <0.2 mU/l and free triiodothyronine (fT3) >6.5 pM. Patients who were pregnant or had been diagnosed with malignancy were excluded. Diagnoses were based on serum levels of TSH and THs, presence of thyroid antibodies, and thyroid gland enlargement. Seventeen patients were diagnosed as having Grave’s disease; 16 of these were treated with tiamazol (Thacapzol) and levothyroxine, and 1 received radioiodine treatment and levothyroxine. One patient was diagnosed as having toxic uninodular goiter and was treated with radioiodine. Two patients were diagnosed as having thyroiditis with transient nodular thyrotoxicosis; they became euthyroid without medical treatment. Blood samples were collected between 08:30 and 09:00 AM after overnight fast on two occasions: before start of treatment and when serum fT3 was normalized (3.0–6.5 pM). The interval between the samplings ranged between 4 and 25 weeks (mean ± SD, 14 ± 6 weeks). In the second study (b), samples were obtained from 14 healthy volunteers (7 women and 7 men) between 25 and 55 years old (mean ± SD, 41 ± 11 years), and with BMI between 22 and 29 kg/m^2^ (mean ± SD, 26 ± 3 kg/m^2^). They had been included in a study evaluating a potential drug interaction between eprotirome and warfarin using a double-blind crossover design (KBT011; Eudra CT 2011-003029-92). Eprotirome is a liver-selective TH receptor agonist that has been tested in human hypercholesterolemia (10–12). Despite promising results in early trials, the development program for eprotirome was discontinued in 2012 due to a toxicology study that revealed cartilage damage in dogs after long-term exposure. Samples taken after 14 days of treatment with 100 µg/day of eprotirome (Karo Bio AB, Sweden) were compared with samples obtained prior to treatment or after a washout period of 14 days after the last dose.

### Body composition

Body weight and composition were measured using a bioelectrical impedance scale (TBF-305; Umedico AB, Sweden).

### THs, lipids, and glucose

Serum levels of fT3, free thyroxine (fT4), TSH, insulin, and plasma levels of total cholesterol, triglycerides, and glucose were measured using a MODULAR ANALYTICS P170/P800 (Roche/Hitachi). Serum levels of cholesterol and triglycerides within VLDL, LDL, and HDL fractions, and glycerol, were measured by fast protein LC (13). For all assays, kits from Roche Diagnostics GmbH (Mannheim, Germany) were used. In eprotirome-treated subjects, insulin levels were measured using ELISA kits (Mercodia AB, Uppsala, Sweden). Serum levels of sex hormone binding globulin (SHBG) were assayed using ELISA kits (SHBG, MX52011; IBL International GmbH, Hamburg, Germany) according to the manufacturer’s instructions. Serum levels of FFAs were measured using kits from Kamiya Biomedical Co. (Seattle, WA) and a Tecan Infinite M200.

### Apos

Serum levels of apoAI (KAI-002), AII (KAI-003), B (KAI-004), CII (KAI-005), and CIII (KAI-006) were determined using immunoturbidimetric assays (Kamiya Biomedical Co.). Serum levels of apoAIV were measured using ELISA kits from Millipore (EZHAP0A4-73K; Billerica, MA). All analyses were carried out in duplicate following the manufacturers’ instructions. Serum lipoprotein(a) [Lp(a)] levels were determined in duplicate samples with an immunoturbidimetric assay using kits from DiaSys Diagnostic Systems GmbH [Lp(a) 21 FS; Holzheim, Germany] and a Response 910 analyzer.

### PCSK9 and FGF19/21

ELISA kits were used to determine serum levels of PCSK9 (CY-8079; CycLex Co. Ltd., Nagano, Japan), FGF19, and FGF21 (DF1900 and DF2100, respectively; R and D Systems Europe Ltd., Abingdon, United Kingdom). All analyses were carried out following the manufacturers’ instructions.

### Bile acid synthesis

In patients, serum levels of 7α-hydroxy-4-cholestene-3-one (C4), a marker of bile acid synthesis (14–17), were assayed in duplicate samples as described (14) and normalized for plasma total cholesterol levels (18). In healthy volunteers, serum levels of the marker 7α-hydroxycholesterol (19) were assayed as described and normalized for plasma cholesterol (20).

### Cholesterol synthesis

Serum levels of the cholesterol synthesis marker lathosterol (21–24) were assayed in the hyperthyroid patients as described (21) and in eprotirome-treated subjects as described (25). Serum levels of lathosterol were normalized for plasma cholesterol.

### Dietary cholesterol absorption

In patients, serum levels of campesterol and sitosterol were determined using GC-MS in duplicate samples as described (7) and in eprotirome-treated subjects as described (26). Serum levels of plant sterols were normalized for plasma cholesterol.

### Serum bile acids

Serum levels of chenodeoxycholic acid (CDCA), cholic acid (CA), deoxycholic acid (DCA), and their amino acid conjugates, were assayed using 250 µl of serum in duplicate samples. Acetonitril was added to samples, which were then centrifuged at 13,000 *g* for 15 min. The upper phase was collected and dried under nitrogen before being dissolved in methanol and analyzed by LC/MS/MS using D_4_-bile acids as internal standards. Bile acids in samples from subjects treated with eprotirome were analyzed as described (27).

### Statistics

Diagrams show individual data, and horizontal bars represent mean levels. Two-tailed Wilcoxon matched-pairs test was used to test significance of differences. Correlations were tested by the Spearman rank correlation coefficient. Significance threshold was set at *P* < 0.05. GraphPad Prism 5.0 Software was used.

### Study approval

The studies were approved by the Ethics Committee at the Karolinska Institute, Stockholm, Sweden, and by the Capenhurst Independent Research Ethics Committee, Capenhurst, United Kingdom, respectively. All participants gave their informed written consent.

## RESULTS

### TH levels and body composition in hyperthyroidism

**Fig. 1.**
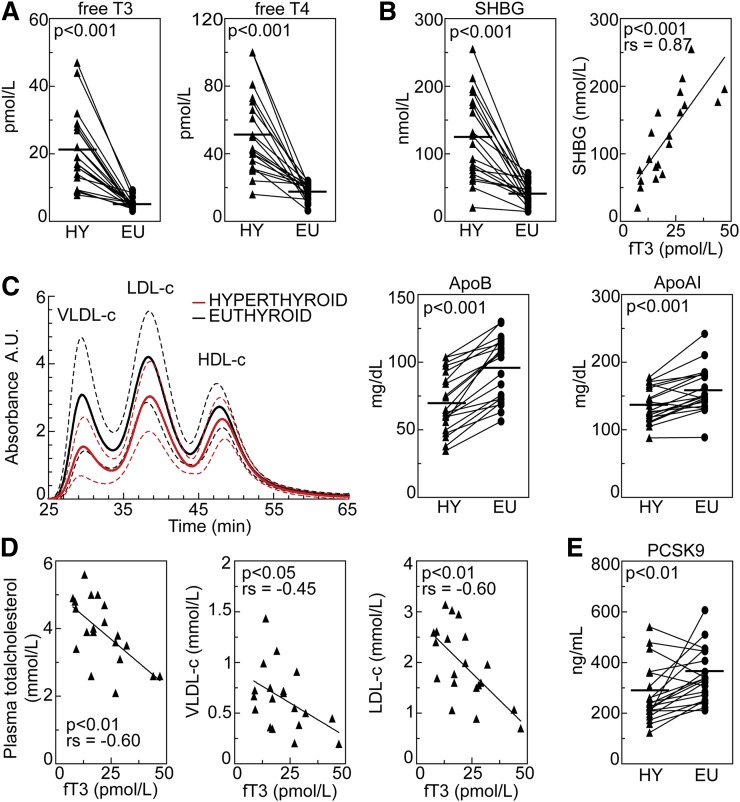
Hyperthyroidism reduces lipoprotein cholesterol, apoB, and PCSK9 levels. Serum levels of fT3 and fT4 (A) in 20 patients before start of treatment in the HY and after clinical normalization in the EU. Serum levels of SHBG reflecting the impact of hyperthyroidism on the liver and correlation between SHBG and fT3 levels in the HY (B). Cholesterol content of specific lipoprotein fractions in the HY (red line) and EU (black line); dotted lines represent SD. Serum levels of apoB and apoAI (C). Correlations between plasma total cholesterol, VLDL-cholesterol, and LDL-cholesterol and fT3 levels in the HY (D). Serum levels of circulating PCSK9 (E). Horizontal bars represent mean values.

In the hyperthyroid state (HY), the increased serum levels of fT3 and fT4 showed a wide variation, averaging 21 ± 11 pM and 52 ± 24 pM, respectively (**Fig. 1A**). The serum level of TSH was suppressed (<0.02 mU/l) in all patients and was increased following treatment, ranging from 0.04 to 7.4 mU/l. In the euthyroid state (EU), fT3 and fT4 were normalized, with mean levels of 5.1 ± 1.6 pM and 18 ± 5.0 pM, respectively. The influence of TH on the liver was reflected by a >2-fold increase in SHBG serum levels, which correlated strongly with free TH levels (Fig. 1B). Body weight and BMI of patients were lower in the HY due to a lower fat mass, whereas lean body mass and body water were unaltered (supplementary Table I).

### Hyperthyroidism lowers lipoprotein cholesterol, apoB, and Lp(a) levels

Plasma total cholesterol was reduced by 28% in hyperthyroidism. The cholesterol content in the VLDL, LDL, and HDL fractions was 48%, 28%, and 15% lower, respectively. Also, serum apoB and apoAI levels were lower, 27% and 14%, respectively (Fig. 1C and supplementary Table I). Plasma total cholesterol, VLDL-cholesterol, and LDL-cholesterol levels correlated inversely with serum levels of fT3 (Fig. 1D) and fT4 in hyperthyroidism, while there was no correlation between HDL-cholesterol and free TH levels. In addition, apoB, but not apoAI, correlated with fT3 levels (*r_s_* = −0.59; *P* < 0.01). No correlations with free TH levels were seen in the EU. Serum Lp(a) levels were 26% lower in hyperthyroidism (supplementary Table I).

### Circulating PCSK9 levels are reduced in hyperthyroidism

PCSK9 regulates hepatic LDLR numbers by disrupting their intracellular recycling, and high plasma PCSK9 levels are thus linked to high LDL-cholesterol and vice versa (8, 28, 29). In hyperthyroidism, serum PCSK9 levels were 22% reduced (Fig. 1E). Although there was no correlation between serum PCSK9 and TH levels, there were clear positive correlations between PCSK9 and plasma total cholesterol and LDL-cholesterol in hyperthyroidism (*r_s_* = 0.48 and *r_s_* = 0.46, respectively; *P* < 0.05). A similar correlation between PCSK9 and LDL-cholesterol levels was also present in the EU (*r_s_* = 0.52; *P* < 0.05).

### Hyperthyroidism does not influence lipoprotein triglycerides but increases peripheral lipolysis

Plasma total triglycerides were unaltered in hyperthyroidism, as was the triglyceride content of specific lipoprotein fractions. Serum levels of FFAs and glycerol were 19% and 35% higher, respectively (supplementary Table I). Irrespective of thyroid state, neither plasma triglycerides, FFAs, nor glycerol correlated with free TH levels. Serum levels of the intestinally derived apoAIV (30) were 19% higher in hyperthyroidism. Serum levels of apoCII were unaltered, while those of apoCIII and apoAII were 15% and 9% lower, respectively (supplementary Table I).

### Hyperthyroidism does not influence serum FGF21, insulin, or glucose levels

FGF21 is a metabolic regulator, with positive impact on glucose and lipid homeostasis when administered to animals (9). In mice, administration of TH increases FGF21 serum levels (31). However, in humans, FGF21 serum levels were unaltered in hyperthyroidism, as were insulin and glucose levels (supplementary Table I).

### Hyperthyroidism increases bile acid synthesis and lowers circulating FGF19, while cholesterol synthesis is unaltered

**Fig. 2.**
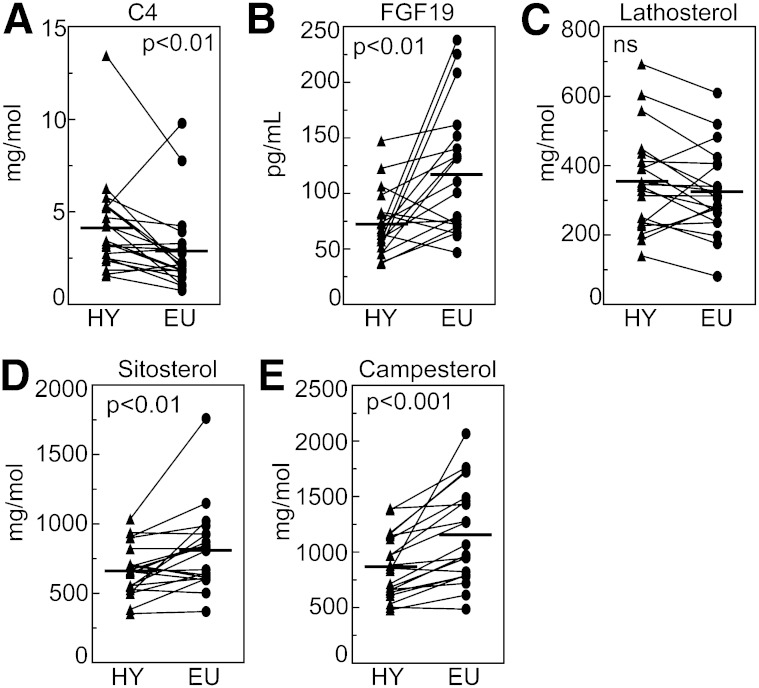
Hyperthyroidism stimulates bile acid synthesis and reduces FGF19 and intestinal absorption of dietary cholesterol. Serum levels of C4 (A), FGF19 (B), lathosterol (C), and plant sterols sitosterol and campesterol (D, E) in 20 hyperthyroid patients before start of treatment in HY and after clinical normalization in EU. Horizontal bars represent mean values.

In mice, TH promotes bile acid synthesis by stimulating the rate-limiting enzyme, cholesterol 7α-hydroxylase (CYP7A1), via hepatic TH β-receptors (5, 32). The data on bile acid turnover and excretion in humans are limited, and so far not conclusive (33–35). In the present study, serum levels of C4, a metabolite formed in the classical bile acid synthetic pathway that closely reflects CYP7A1 activity and bile acid synthesis (14–17), were 43% higher in hyperthyroidism, showing that bile acid synthesis is stimulated by TH in humans (**Fig. 2A**). This increase in synthesis appeared concomitantly with a 29% reduction of serum FGF19 (Fig. 2B). FGF19 is believed to be secreted from ileal enterocytes in response to farnesoid X receptor (FXR) activation (9) and has been hypothesized to inhibit bile acid synthesis in the liver by suppressing CYP7A1. In line with this concept, there was an inverse correlation between serum levels of FGF19 and C4 in the EU (*r_s_* = −0.46; *P* < 0.05). However, no such relationship was found in hyperthyroidism. Serum levels of lathosterol, a precursor of cholesterol that reflects cholesterol synthesis (21–24), were unaltered in hyperthyroidism (Fig. 2C). This indicates that, unlike what is observed in rodents (5, 36), cholesterol synthesis is not stimulated by TH in humans.

### Hyperthyroidism decreases intestinal absorption of cholesterol

Animal data indicate that TH reduces intestinal absorption of dietary cholesterol (7), which should contribute to lower plasma cholesterol. Because plant sterols and cholesterol share common pathways for uptake into and excretion from enterocytes, serum levels of plant sterols can be used to estimate absorption of dietary cholesterol (23). In hyperthyroidism, serum levels of plant sterols campesterol and sitosterol were lowered by 25% and 18%, respectively, indicating that absorption of dietary cholesterol is reduced by TH also in humans (Fig. 2D, E). However, because the uptake of plant sterols from the intestine competes with the uptake of cholesterol of dietary and biliary origin, their reduced levels may also reflect an increased biliary secretion of cholesterol.

### Hyperthyroidism influences serum bile acid composition and conjugation

Total level of bile acids in serum was unchanged in hyperthyroidism. The proportion of CA was unaltered, while the proportions of CDCA and DCA were 26% higher and 42% lower, respectively (supplementary Table I). The relative amount of conjugated bile acids was 25% higher and correlated positively with fT3 levels (*r_s_* = 0.88; *P* < 0.001). The increased conjugation was the result of a greater amount of taurine conjugated bile acids (+73%); this change also correlated closely with fT3 (*r_s_* = 0.80; *P* < 0.001). Accordingly, the ratio of glycine to taurine conjugated bile acids was 35% lower in hyperthyroidism; again this was strongly correlated with fT3 (*r_s_* = −0.70; *P* < 0.01).

### Liver-selective stimulation of TH receptors by eprotirome reduces lipoprotein cholesterol, Lp(a), and PCSK9 levels

**Fig. 3.**
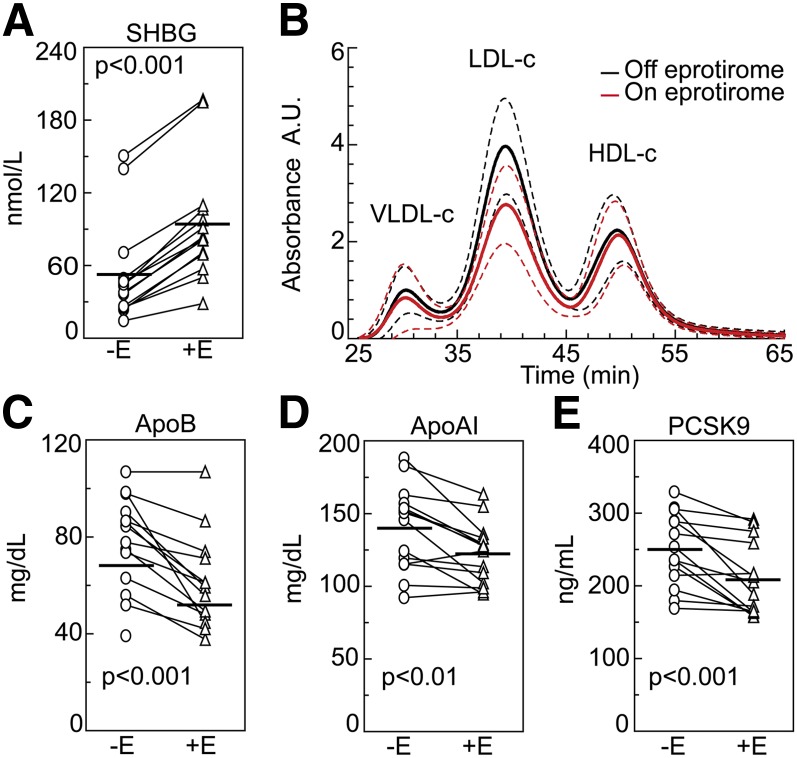
Stimulation of hepatic TH receptors by eprotirome treatment lowers lipoprotein cholesterol, apoB, and PCSK9 levels. Serum levels of SHBG (A) in 14 healthy subjects off (**−**E) and on (**+**E) treatment with the liver-selective thyromimetic eprotirome. Cholesterol content of specific lipoprotein fractions off treatment (black line) and on treatment (red line); dotted lines represent SD (B). Serum levels of apoB (C), apoAI (D), and circulating PCSK9 (E). Horizontal bars represent mean values.

Serum SHBG levels were increased (+80%) by eprotirome treatment, indicating a marked stimulation of hepatic TH receptors (**Fig. 3A**). In similarity to hyperthyroidism, plasma total cholesterol was 21% lower in response to treatment, and VLDL-, LDL-, and HDL-cholesterol were reduced by 20%, 29%, and 10%, respectively (Fig. 3B and supplementary Table II). Eprotirome reduced apoB and apoAI levels by 21% and 13%, respectively (Fig. 3C, D). Also consistent with the findings in hyperthyroidism, eprotirome treatment was associated with markedly reduced (−25%) levels of Lp(a) (supplementary Table II) as well as PCSK9 (−17%) (Fig. 3E).

### Eprotirome reduces lipoprotein triglycerides but does not increase peripheral lipolysis

In contrast to hyperthyroidism, eprotirome treatment lowered plasma total triglycerides. VLDL-, LDL-, and HDL-triglyceride levels were reduced by 35%, 38%, and 46%, respectively. Also in contrast to hyperthyroidism, serum levels of FFAs, glycerol, and apoAII were unaltered in eprotirome-treated subjects (supplementary Table II). ApoAIV levels were also unaltered, in opposition to the decrease in hyperthyroidism and in agreement with the concept that apoAIV is mainly produced by the intestine (30, 37). In similarity with hyperthyroidism, serum levels of apoCII were unaltered, while those of apoCIII were reduced by 26%. Serum FGF21, insulin, and plasma glucose levels were also not altered by eprotirome treatment (supplementary Table II).

### Eprotirome does not substantially influence bile acid or cholesterol synthesis, nor FGF19 levels or cholesterol absorption

**Fig. 4.**
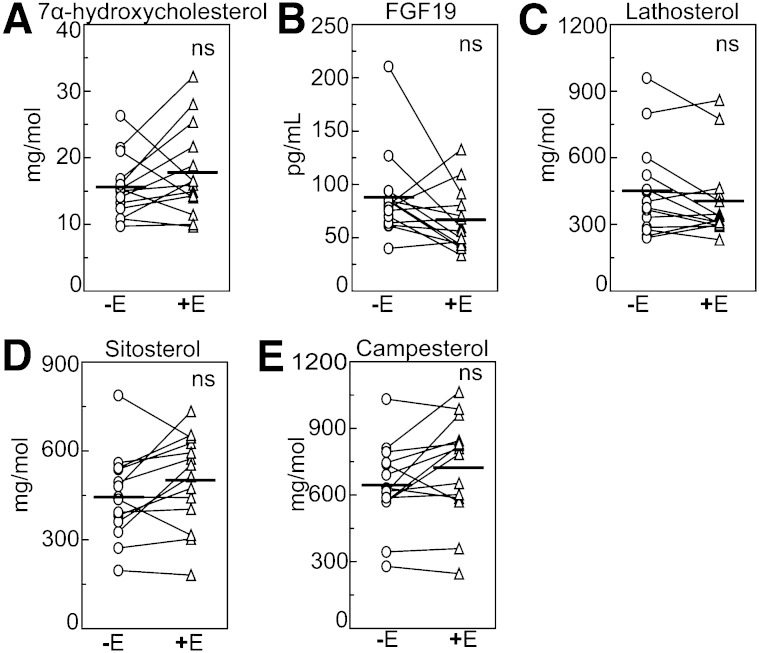
Bile acid synthesis, FGF19, cholesterol synthesis, and intestinal absorption are unaltered by stimulation of hepatic TH receptors by eprotirome treatment. Serum levels of 7α-hydroxycholesterol (A), FGF19 (B), lathosterol (C), and plant sterols sitosterol and campesterol (D, E) in 14 healthy subjects off (−E) and on (+E) treatment with the liver-selective thyromimetic eprotirome. Horizontal bars represent mean values.

In eprotirome-treated subjects, bile acid synthesis was estimated from serum levels of the bile acid precursor 7α-hydroxycholesterol (24). When eprotirome was given at a dose of 100 µg/day, serum levels of 7α-hydroxycholesterol were not significantly changed, nor were those of lathosterol (**Fig. 4A, C**). This indicates that, in contrast to hyperthyroidism, eprotirome at the dose given did not markedly increase bile acid synthesis. Again, in contrast to hyperthyroidism, FGF19 levels were unaltered following eprotirome treatment (Fig. 4B). Total serum bile acids were 19% higher after eprotirome treatment. While the relative amounts of CA and DCA were unaltered, that of CDCA was 17% higher, similar to what was seen in hyperthyroidism (supplementary Table II). Serum levels of plant sterols campesterol and sitosterol were unaltered by eprotirome treatment (Fig. 4D, E) supporting the concept that treatment with a liver-selective thyromimetic does not alter absorption of dietary cholesterol from the intestine.

## DISCUSSION

TH is essential in regulating metabolic rate and lipid homeostasis (1–4, 38). In the present work, studies in how elevated TH levels influence cholesterol and lipoprotein metabolism in humans were performed. By comparing the HY and EU in the same individual, the influence of interindividual genetic variation was reduced, and the wide range in TH levels in hyperthyroid patients provided a possibility to relate metabolic responses to hormone levels. By comparing the responses to hyperthyroidism with those induced in healthy subjects by treatment with the liver-selective TH analog eprotirome, the importance of liver-specific effects of TH in humans was also explored.

First, we could confirm that TH lowers plasma cholesterol in all lipoprotein fractions, and that this depends mainly on TH actions in the liver. The degree of LDL-cholesterol lowering was proportional to free TH levels, and related to the degree of PCSK9 reduction. From previous human studies on lipoprotein kinetics, it is clear that plasma LDL-cholesterol is lowered by TH mainly through stimulation of LDL clearance (39), presumably due to an increased number of hepatic LDLRs. The reductions in LDL-cholesterol and PCSK9 levels were of similar magnitude in both hyperthyroid and eprotirome-treated individuals, indicating that this is a liver-specific action of TH. As predicted from previous data (29, 40), the change in PCSK9 levels in response to TH is compatible with a substantial reduction of LDL-cholesterol. Thus, in addition to transcriptional stimulation of the LDLR gene, the reduced PCSK9 level should contribute substantially to increase the number of hepatic LDLRs in hyperthyroidism. The finding that lathosterol levels were unaltered may indicate that TH partly influences PCSK9 through non-sterol-regulatory element binding protein (SREBP) 2-mediated effects (41). Although the previously discussed results strongly suggest that the liver is a key organ for the changes in plasma cholesterol induced by TH, the possibility of concomitant extrahepatic effects cannot be entirely excluded.

Second, we could establish that TH markedly reduced levels of the atherogenic Lp(a), and that this was also dependent on its hepatic action. How Lp(a) serum levels are regulated in humans is unclear (42), but it is generally acknowledged that hepatic synthesis is important. Inhibition of PCSK9 also lowers Lp(a) (43), indicating that the TH-induced reduction of circulating PCSK9 may be involved in the lowering of Lp(a).

Third, bile acid synthesis, evaluated from measurements of the well-established marker C4 (14), was induced in hyperthyroidism. This occurred without increased cholesterol synthesis, indicating that a net amount of cholesterol is drained from the body. In animal models, TH increases the expression of CYP7A1 (32, 44), and it has been suggested as one of the major mechanisms for lowering plasma cholesterol (32, 45). Due to the complexity of many of the techniques used to assess bile acid turnover in vivo, the extent of human data has been rather limited, and so far inconclusive (33–35, 46). In rodents, TH inhibits the rate-limiting enzyme in CA production, sterol 12α-hydroxylase (CYP8B1), resulting in increased CDCA synthesis (47). The fact that such a change in the relative contribution of CDCA to the circulating bile acid pool was observed in hyperthyroid and in eprotirome-treated subjects indicates that TH also suppresses CYP8B1 in human liver. The increased conjugation of circulating bile acids with taurine is also in agreement with previous work (48), and it will be of interest to analyze if any of the effects of TH can be related to the change in conjugation pattern.

Fourth, serum FGF19 levels were clearly reduced in hyperthyroidism. FGF19 is presumably secreted from the ileum in response to activation of FXR by bile acids (49) and contributes to negative feedback regulation of bile acid synthesis by inhibition of hepatic CYP7A1 (9). The effects of eprotirome on bile acid synthesis and FGF19 were not statistically significant and could indicate that TH has a direct effect on the small intestine, either on bile acid reabsorption or on FGF19 secretion. This interpretation calls for some caution, however, because a higher dose of eprotirome (200 µg/day) has been shown to induce bile acid synthesis in humans (10). Nevertheless, the fact that eprotirome markedly lowered LDL-cholesterol, apoB, and Lp(a) levels demonstrates that these effects are not driven by an induced bile acid synthesis.

Fifth, there was a clear difference between the effect of hyperthyroidism and of liver-selective TH receptor activation on plasma triglyceride levels. Whereas eprotirome reduced triglycerides in all lipoproteins, there were no such changes in hyperthyroidism. There was evidence of stimulated peripheral lipolysis, with elevated levels of FFA and glycerol, in hyperthyroidism, but not in eprotirome-treated subjects. Levels of apoB and apoCIII, mainly produced in the liver, showed similar changes in the two TH exposure models, whereas apoAII and apoAIV that are predominantly from the small intestine were increased in hyperthyroidism but not eprotirome-treated subjects. One explanation may be that the hepatic effects of TH that probably include suppression of the master regulator of lipid synthesis, SREBP1c (45), are counterbalanced by an increased influx of FFAs from peripheral tissues exposed to TH in hyperthyroidism.

Sixth, neither hyperthyroidism nor eprotirome treatment had an effect on the circulating FGF21. This finding contrasts what has been reported for mice, where TH induces hepatic FGF21 gene expression and serum levels dose dependently (31). The role of FGF21 in metabolic regulation is still unclear, and there are also species differences regarding FGF21 in metabolic regulation (9). Because circulating FGF21 has been proposed to be regulated by FFA levels in humans (50), it is interesting to note the lack of effect on FGF21 in the hyperthyroid patients, despite their increased serum FFA levels.

Finally, HDL-cholesterol and apoAI levels were reduced in hyperthyroidism and by eprotirome treatment. In epidemiological studies, low HDL-cholesterol and apoAI are associated with an enhanced risk of coronary heart disease (51). However, animal studies show that HDL-mediated efflux of cholesterol from peripheral cells to the liver and its subsequent elimination may be stimulated by TH or thyromimetics (45, 52, 53). Thus, a decreased level of HDL-cholesterol may actually reflect a stimulated turnover of peripherally derived cholesterol, indicating the presence of an “antiatherogenic,” rather than an “atherogenic,” state.

**Fig. 5.**
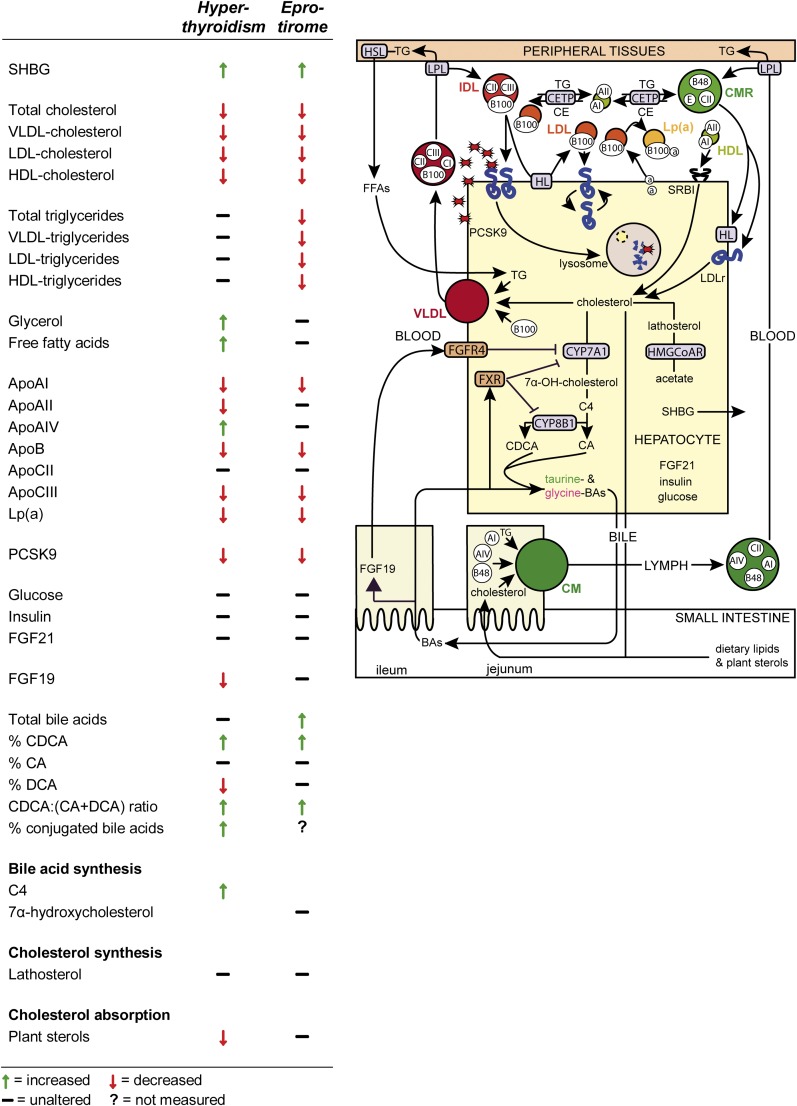
Summary of the effects of hyperthyroidism and eprotirome on serum markers and metabolites in lipid metabolism. 7α-OH-cholesterol, 7α-hydroxycholesterol; BAs, bile acids; CE, cholesteryl ester; CETP, cholesteryl ester transfer protein; CM, chylomicron; CMR, chylomicron remnant; HMG-CoAR, HMG-CoA reductase; HSL, hormone sensitive lipase; SRBI, scavenger receptor class B type I.

In conclusion, TH exerts several important effects on cholesterol and lipoprotein metabolism in humans, as outlined in **Fig. 5**. Hepatic actions of TH are responsible for most of the positive effects including lowering of PCSK9 and LDL-cholesterol levels, as well as reduction of apoB and Lp(a). Bile acid synthesis is stimulated in hyperthyroidism, but this does not appear critical for lowering LDL-cholesterol. Intestinal actions of TH for its pronounced effects on bile acid synthesis and cholesterol absorption seem more important than previously recognized. Selective activation of TH receptors in the liver lowers plasma triglycerides, whereas a concomitant stimulation of peripheral lipolysis during hyperthyroidism counteracts this action.

## Supplementary Material

Supplemental Data
