# Supplementary material for: Thyroid hormone reduces PCSK9 and stimulates bile acid synthesis in humans
Source: J Lipid Res. 2014 Nov;55(11):2408–15. doi: 10.1194/jlr.M051664 (PMC4617142; doi:10.1194/jlr.M051664)
Supplement: Supplemental Data [file supp_55_11_2408__index.html]

Thyroid hormone reduces PCSK9 and stimulates bile acid synthesis in humans — Thyroid hormone reduces PCSK9 and stimulates bile acid synthesis in humans — Supplemental Data 

# Thyroid hormone reduces PCSK9 and stimulates bile acid synthesis in humans

## Supplemental Data

**Files in this Data Supplement:**

- Supplemental Table 1 and 2 - Supplemental Table I. Body composition and levels of lipids, apolipoproteins, FGF21, insulin, glucose and bile acids in serum or plasma in 20 hyperthyroid patients before and after clinical normalization. Supplemental Table II. Levels of lipids, apolipoproteins, FGF21, insulin, glucose and bile acids in serum or plasma in the same 14 healthy subjects off and on treatment with the liver-selective TH analog eprotirome.
