# Supplementary material for: Thyroid hormone reduces PCSK9 and stimulates bile acid synthesis in humans
Source: J Lipid Res. 2014 Nov;55(11):2408–15. doi: 10.1194/jlr.M051664 (PMC4617142; doi:10.1194/jlr.M051664)
Supplement: Supplemental Data [file supp_M051664_jlr.M051664-1.pdf]

Supplemental Table I. Body composition and levels of lipids, apolipoproteins, FGF21, insulin, glucose and bile acids in serum or plasma in 20 hyperthyroid patients before and after clinical normalization.

	**HYPERTHYROID mean ±SD**	**EUTHYROID mean ±SD**	**p value**	**% change**
Body weight, *kg*	63.6 ±10.2	66.4 ±9.95	<0.001	-4
BMI, *kg/m*^{2}	22.8 ±3.60	23.8 ±3.37	<0.001	-4
Total body water, *kg*	33.7 ±6.31	33.0 ±6.12	ns	
Lean body mass, *kg*	46.1 ±8.61	45.0 ±8.32	ns	
Body fat mass, *kg*	17.8 ±7.64	21.3 ±8.34	<0.001	-16
Total cholesterol, *mmol/L*	3.90 ±0.97	5.40 ±1.17	<0.001	-28
VLDL-cholesterol, *mmol/L*	0.64 ±0.31	1.23 ±0.60	<0.001	-48
LDL-cholesterol, *mmol/L*	1.70 ±0.58	2.35 ±0.71	<0.001	-28
HDL-cholesterol, *mmol*/L	1.50 ±0.40	1.77 ±0.37	<0.01	-15
Total triglycerides, *mmol/L*	1.04 ±0.47	1.01 ±0.41	ns	
VLDL-triglycerides, *mmol/L*	0.34 ±0.19	0.38 ±0.19	ns	
LDL-triglycerides, *mmol/L*	0.28 ±0.15	0.28 ±0.14	ns	
HDL-triglycerides, *mmol/L*	0.19 ±0.09	0.18 ±0.08	ns	
Glycerol, *mmol/L*	0.23 ±0.11	0.17 ±0.06	<0.05	35
Free fatty acids, *mmol/L*	1.17 ±0.35	0.98 ±0.36	<0.05	19

Supplemental Table I *continued*.

	HYPERTHYROID mean ±SD	EUTHYROID mean ±SD	p value	% change
Lp(a), *nmol/L*	28.9 ±24.2	38.9 ±34.3	<0.001	-26
ApoAIV, *mg/dL*	42.5 ±17.2	35.8 ±18.6	<0.05	19
ApoCII, *mg/dL*	4.37 ±0.96	4.71 ±1.27	ns	
ApoCIII, *mg/dL*	8.71 ±2.49	10.2 ±3.11	<0.01	-15
ApoAII, *mg/dL*	33.2 ±6.63	36.4 ±7.00	<0.01	-9
FGF21, *pg/mL*	191 ±107	176 ±124	ns	
Insulin, *pmol/L*	50.9 ±24.2	50.4 ±19.9	ns	
Glucose, *mmol/L*	5.36 ±0.72	5.23 ±0.70	ns	
Total BAs, *µmol/L*	2.14 ±2.12	2.35 ±2.55	ns	
CA, *%*	19.8 ±8.42	18.1 ±6.44	ns	
CDCA, *%*	61.0 ±15.0	48.6 ±20.9	<0.01	26
DCA, *%*	19.2 ±16.4	33.2 ±22.6	<0.01	-42
Conjugated BAs, *%*	82.3 ±17.8	65.8 ±19.3	<0.01	25
Taurine conjugated BAs, *%*	22.0 ±14.0	12.7 ±9.60	<0.05	73

Lp(a); lipoprotein(a), FGF21; fibroblast growth factor 21, BAs; bile acids, CA; cholic acid, CDCA; chenodeoxycholic acid, DCA; deoxycholic acid

Supplemental Table II. Levels of lipids, apolipoproteins, FGF21, insulin, glucose and bile acids in serum or plasma in the same 14 healthy subjects off and on treatment with the liver-selective TH analog eprotirome.

	**Off eprotirome mean ±SD**	**On eprotirome mean ±SD**	**p value**	**% change**
Total cholesterol, *mmol/L*	4.5 ±0.67	3.6 ±0.60	<0.001	-21
VLDL-cholesterol, *mmol/L*	0.45 ±0.27	0.36 ±0.29	<0.05	-20
LDL-cholesterol, *mmol/L*	2.39 ±0.52	1.70 ±0.43	<0.01	-29
HDL-cholesterol, *mmol*/L	1.67 ±0.39	1.50 ±0.38	<0.01	-10
Total triglycerides, *mmol/L*	1.50 ±1.70	0.97 ±0.65	<0.05	-35
VLDL-triglycerides, *mmol/L*	0.72 ±0.91	0.47 ±0.50	<0.05	-35
LDL-triglycerides, *mmol/L*	0.42 ±0.38	0.26 ±0.11	<0.01	-38
HDL-triglycerides, *mmol/L*	0.24 ±0.30	0.13 ±0.05	<0.01	-46
Glycerol, *mmol/L*	0.16 ±0.17	0.13 ±0.07	ns	
Free fatty acids, *mmol/L*	0.69 ±0.35	0.85 ±0.45	ns	
Lp(a), *nmol/L*	38.3 ±39.0	28.8 ±32.1	<0.01	-25
ApoAIV, *mg/dL*	49.7 ±12.9	51.1 ±15.2	ns	
ApoCII, *mg/dL*	4.20 ±1.30	4.09 ±1.41	ns	
ApoCIII, *mg/dL*	10.3 ±5.41	7.64 ±1.97	<0.01	-26
ApoAII, *mg/dL*	37.3 ±5.80	36.5 ±4.70	ns	

Supplemental Table II *continued*.

	Off eprotirome mean ±SD	On eprotirome mean ±SD	p value	% change
FGF21, *pg/mL*	170 ±89.0	153 ±129	ns	
Insulin, *pmol/L*	34.8 ±14.4	38.6 ±15.3	ns	
Glucose, *mmol/L*	5.07 ±0.55	5.08 ±0.46	ns	
Total BAs, *µmol/L*	0.77 ±0.57	0.92 ±0.47	<0.05	19
CA, *%*	19.8 ±7.23	18.5 ±6.62	ns	
CDCA, *%*	37.7 ±11.5	44.0 ±8.41	<0.01	17
DCA, *%*	42.5 ±14.9	37.6 ±11.1	ns	

Lp(a); lipoprotein(a), FGF21; fibroblast growth factor 21, bile acids; BAs, CA; cholic acid, CDCA; chenodeoxycholic acid, DCA; deoxycholic acid
